# Hazard Ranking Methodology for Assessing Health Impacts of Unconventional Natural Gas Development and Production: The Maryland Case Study

**DOI:** 10.1371/journal.pone.0145368

**Published:** 2016-01-04

**Authors:** Meleah D. Boyle, Devon C. Payne-Sturges, Thurka Sangaramoorthy, Sacoby Wilson, Keeve E. Nachman, Kelsey Babik, Christian C. Jenkins, Joshua Trowell, Donald K. Milton, Amir Sapkota

**Affiliations:** 1 Maryland Institute for Applied Environmental Health, School of Public Health, University of Maryland, College Park, Maryland, United States of America; 2 Department of Anthropology, College of Behavioral and Social Sciences, University of Maryland, College Park, Maryland, United States of America; 3 Center for a Livable Future, Johns Hopkins Bloomberg School of Public Health, Baltimore, Maryland, United States of America; Centro de Investigacion Cientifica y Educacion Superior de Ensenada, MEXICO

## Abstract

The recent growth of unconventional natural gas development and production (UNGDP) has outpaced research on the potential health impacts associated with the process. The Maryland Marcellus Shale Public Health Study was conducted to inform the Maryland Marcellus Shale Safe Drilling Initiative Advisory Commission, State legislators and the Governor about potential public health impacts associated with UNGDP so they could make an informed decision that considers the health and well-being of Marylanders. In this paper, we describe an impact assessment and hazard ranking methodology we used to assess the potential public health impacts for eight hazards associated with the UNGDP process. The hazard ranking included seven metrics: 1) presence of vulnerable populations (e.g. children under the age of 5, individuals over the age of 65, surface owners), 2) duration of exposure, 3) frequency of exposure, 4) likelihood of health effects, 5) magnitude/severity of health effects, 6) geographic extent, and 7) effectiveness of setbacks. Overall public health concern was determined by a color-coded ranking system (low, moderately high, and high) that was generated based on the overall sum of the scores for each hazard. We provide three illustrative examples of applying our methodology for air quality and health care infrastructure which were ranked as high concern and for water quality which was ranked moderately high concern. The hazard ranking was a valuable tool that allowed us to systematically evaluate each of the hazards and provide recommendations to minimize the hazards.

## Introduction

While increasing domestic production of natural gas provides economic growth and jobs, there is concern that new unconventional natural gas development and production (UNGDP) extraction technologies could negatively impact public health, the environment, and natural resources. The Marcellus Shale gas formation, found beneath the surface of Pennsylvania, Ohio, West Virginia, New York and Western Maryland contains more than 410 trillion cubic feet of natural gas and is a major target for UNGDP [1]. Natural gas development and production has begun in several areas, including Ohio, Pennsylvania, and West Virginia, whereas in Maryland, the Government established a Marcellus Shale Safe Drilling Initiative to evaluate the potential economic, environmental, and public health impacts prior to deciding whether and how to allow development of the State’s natural gas resources.

There has been increasing concern among the public health community that the quick spread of UNGDP has left little time for a thorough evaluation of the health impacts [2–4]. While there are few epidemiologic studies on the health impacts associated with natural gas development, recently published studies and state-funded health assessments have begun to illuminate the major hazards and exposure pathways that potentially lead to adverse health effects. The hazards of concern include air, water, and soil quality, environmental noise, earthquakes, exposure to toxic chemicals, occupational health, and secondary impacts including mental health and disruption of the social fabric in impact communities [4–9].

On June 6, 2011, then Governor Martin O'Malley issued Executive Order 01.01.2011.11, establishing the Marcellus Shale Safe Drilling Initiative (Initiative). The Initiative’s purpose was to assist state policymakers and regulators in determining whether and how gas production from the Marcellus Shale and other shale formations in Maryland can be accomplished without unacceptable risks to public health, safety, the environment, and natural resources. On October 18, 2013, the Maryland Department of Health and Mental Hygiene (DHMH) signed a memorandum of understanding (MOU) with the Maryland Institute of Applied Environmental Health (MIAEH) at the University of Maryland, College Park to evaluate the potential public health impacts associated with drilling in the Marcellus Shale in Maryland.

We conducted this public health study using the Health Impact Assessment (HIA) framework to inform the Marcellus Shale Safe Drilling Initiative Advisory Commission, State legislators and the Governor about potential health impacts associated with UNGDP related activities. HIA combines stakeholder input, quantitative and qualitative analysis methods, and a variety of data sources to determine the potential health effect of a proposed policy, plan, program, or project and then provides recommendations for mitigating the identified impacts [10]. HIA typically consists of 6 steps:

1. Screening: Initial step to determine the need for HIA.
2. Scoping: With community input, identify the most important hazard and health impacts on which to.
3. Assessment: Analyze the baseline characteristics of the population and provide anticipated potential effects.
4. Recommendations: Based on the assessment, develop recommendations for minimizing health effects, and approaches for monitoring.
5. Reporting: Prepare a report for the decision makers, disseminate the findings and recommendations to all the stakeholders including community members.
6. Monitoring and Evaluation of the HIA Process: Evaluate if the HIA process helped the decision making process.

In this manuscript, we describe a hazard ranking methodology we used to assess the potential public health impacts associated with the UNGDP process. We also provide three illustrative examples of applying the methodology which were reported in *Potential Public Health Impacts of Natural Gas Development and Production in the Marcellus Shale in Western Maryland* [11]. For complete list of hazards and their respective rankings, we refer the readers to our full report available at: http://www.marcellushealth.org/.

## Materials and Methods

### Study Area

**Fig 1:**
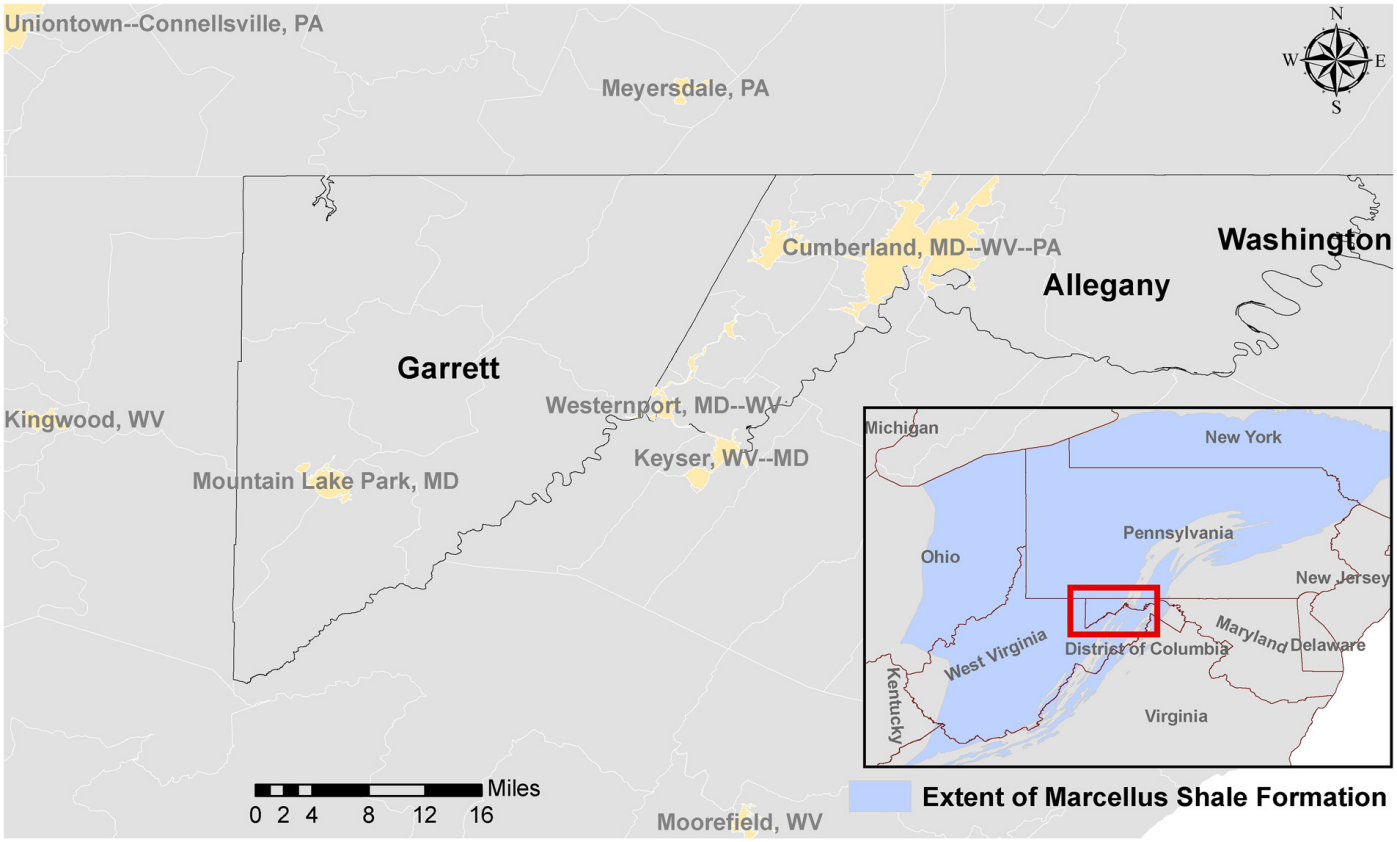
Extent of the Marcellus Shale in Garret and Allegany Counties in Western Maryland.

The study focused on two counties in Western Maryland (Fig 1). Allegany County (population 75,087) covers 424.16 square miles in the northwestern part of Maryland, and Garrett County (population 30,097) covers 647.10 square miles in the western part of the state. Garrett County has over 76,000 acres of parks, lakes, and publicly accessible forestland. Nicknamed Maryland’s “Mountaintop Playground," the county has the state’s highest elevation at 3,360 feet, as well as its largest inland body of water (Deep Creek Lake). Garrett County is home to the state's only sub-arctic wetlands and is the only county in the state that currently produces natural gas (by conventional methods). There are approximately 153 churches, 87 schools, and 3 hospitals in both counties.

### Scoping

We conducted a scoping process to determine the overarching aims and objectives of the HIA, which hazards and health impacts to evaluate, and a research strategy and methods for analysis and conducting literature reviews. The scoping process included input from a wide range of stakeholders, through public meetings and a review of 113 public comments submitted to the Maryland Department of Environment on the Marcellus Shale Safe Drilling Initiatives Best Practices Report. The stakeholder input and public comments were used along with a review of the issues identified in the DHMH MOU to develop a list of specific topics to be investigated as part of this HIA.

On September 24, 2013 and October 5, 2013, we held two public engagement meetings in Western Maryland to discuss community concerns about natural gas exploration and development. Both meetings were open to the public and were advertised widely through press releases, radio and newspaper announcements, email blasts, and word of mouth. Attendees included community members, representatives from local community-based organizations, and health department and other government officials. The meetings were audio recorded and transcribed, and attendees were asked to submit notecards with additional concerns not discussed during the meeting. Based on comments received and expressed in the meetings, ten key themes emerged in the order of number of concerns expressed: water quality, zoning, baseline health assessment, secondary impacts, economic impacts, climate change/weather, air quality, populations of concern, occupational impacts, healthcare infrastructure, and benefits. These themes served as the basis for identifying the hazards that were ultimately reviewed.

### Literature Review

We conducted an extensive review of the peer-reviewed literature, as well as reports from federal and state governments and nonprofit organizations using ISI Web of Knowledge (www.isiknowledge.com) and PubMed (http://www.ncbi.nlm.nih.gov/pubmed) between October 2013 and May 2014. Additional publications were identified based on communication with experts, references cited within the published articles, and the ‘citation track’ feature available from the ISI Web of Knowledge. The search terms included fracking’ OR ‘hydraulic fracturing’ OR ‘natural gas’ OR ‘unconventional natural gas’ OR ‘Marcellus shale’ AND ‘air quality’ OR ‘air pollution’ OR ‘water quality’ OR ‘water pollution’ OR ‘radiation’ OR ‘health effects’ OR ‘adverse health outcomes’ OR ‘public health’. Additional searches were conducted using Physicians Scientists and Engineers for Healthy Energy (PSE) Citation Database on Shale Gas and Tight Oil Development (http://www.psehealthyenergy.org/site/view/1180). We also used Google and Google Scholar to search for government reports and reports from non-governmental organizations. Finally, we considered additional reports and articles submitted to us from the community, industry groups, and environmental advocacy organizations. In total, we reviewed 312 peer-reviewed papers, government reports, and non-governmental reports.

### Site Visits

We visited communities where UNGDP was currently underway to directly observe the process. We conducted two focus groups with communities in Doddridge County, West Virginia to gain further insight about communities currently impacted by UNGDP [12]. We held discussions with representatives from the American Petroleum Institute to better understand the UNGDP process as well as recent changes and technological advances that may directly impact the potential hazards. Finally, we conducted noise monitoring in Doddridge County, West Virginia, where UNGDP is actively underway. This study was approved by the University of Maryland Institutional Review Board (UMD-IRB). Prior to data collection, we received signed informed consent from all individuals participating in the focus group. Informed consents were also obtained from the property owners prior to conducting noise monitoring.

### Synthesis

We synthesized all the comments received during the scoping process, literature review, and site visits to identify the specific hazards to be addressed by the HIA. The hazards were organized into eight categories: 1) air quality, 2) water quality (including water quality, soil quality, and naturally occurring radiological materials), 3) noise, 4) earthquakes, 5) social determinants of health (e.g. crime, injuries, mental health, sexually transmitted infections, and substance abuse), 6) occupational health, 7) healthcare infrastructure, and 8) cumulative exposures and risk.

### Impact Assessment

Our impact assessment focused on the eight hazards that would be concentrated in and/or unique to the Garrett and Allegany County populations living and working near the sites of shale gas development. To provide a succinct overview of the impact of UNGDP associated hazards on public health, we conducted a qualitative assessment of potential impacts as described below. This approach also enabled us to provide an assessment despite limited data on health and environmental effects of UNGDP.

We modified a hazard ranking/scoring system previously described by Witter and colleagues by incorporating information we obtained through out literature review and synthesis of the scoping and focus groups [8]. To evaluate hazards, Witter and colleagues considered several key elements including duration and frequency of exposure, the likelihood and severity of health effects, the geographic extent, and the impact on vulnerable populations. Their approach also included the direction of health effects—positive vs. negative. Each hazard was given a positive (+) or negative (-) numbered score, ranging from -6 to 15.

There are two main differences between our hazard ranking methodology and the one used by Witter and colleagues. First, we added effectiveness of setback because of the prominence of this proposed regulatory approach as outlined by the state of Maryland in the Marcellus Shale Safe Drilling Initiative Study, Part II Best Practices [13]. Setback is defined as the distance between a building (ex: private residence, school, church) or natural resource (ex: river, wetlands, irreplaceable natural areas) and UNGDP activity including location of well pads, pipelines, access roads, or compressor stations [13]. Recognizing that not all hazards will be mitigated by the setback regulations, we wanted to integrate evaluation of whether or not such regulatory approach, if enacted, would mitigate exposure into our overall assessment of the hazards. Second, we added public health impact, which employs a color-coded system to rank the potential impacts of each hazard on public health. These color coded schemes were generated based on the overall sum of scores for each hazard and were added to make it easier for the general public to understand the results.

The modified metrics included in our evaluation were: 1) presence of vulnerable populations (e.g. children under the age of 5, individuals over the age of 65, property owners who only hold legal or equitable title to the land surface and not the mineral resources underground, also known as “surface owners”, proximity to well pads or other UNGDP related infrastructure), 2) duration of exposure, 3) frequency of exposure, 4) likelihood of health effects, 5) magnitude/severity of health effects, 6) geographic extent, and 7) effectiveness of the setback.

## Results

Our final impact assessment methodology consisted of a scoring and qualitative ranking system across eight hazards: 1) air quality, 2) water quality (including water quality, soil quality, and naturally occurring radiological materials), 3) noise, 4) earthquakes, 5) social determinants of health, 6) occupational health, 7) healthcare infrastructure, and 8) cumulative exposures and risk. Each of the hazards are assessed according to criteria in Table 1 and assigned a score. We included an “unknown” category in our ranking system and assigned a score of 1 instead of 0 to show that lack of data and information does not indicate that there is no concern. While we did not deliberately add weight to any of the metrics, there was some inadvertent weighting due to difference in gradations for the criteria. For example, duration of exposure, likelihood of health effects, and magnitude/severity of health effects hold more weight than the other binary criteria. Adding weight to these factors allowed us to put slightly more emphasis on the health effects associated with each of the hazards.

**Table 1:** Description of the evaluation criteria used for hazard ranking.

Evaluation Criteria	Result	Score	Description
Presence of vulnerable populations	No	1	Affects all populations equally
	Yes	2	Disproportionately affects vulnerable populations
Duration of exposure	Short	1	Lasts less than 1 month
	Medium	2	Lasts at least one month but less than one year
	Long	3	Lasts one year or more
Frequency of exposure	Infrequent	1	Occurs sporadically or rarely
	Frequent	2	Occurs constantly, recurrently
Likelihood of health effects	Unlikely	0	Prior evidence suggests exposure is not related to adverse health outcomes
	Unknown	1	Evidence is inconclusive/insufficient data
	Possible	2	Prior evidence suggests exposures may be associated with adverse health outcomes
	Likely	3	Prior evidence suggests similar exposures to be associated with adverse health outcomes
Magnitude/severity of health effects	None	0	No adverse health effects
	Unknown	1	Evidence inconclusive/insufficient data
	Low	2	Causes health effects that can be quickly and easily managed, do not require medical treatment
	Medium	3	Causes health effects that necessitate treatment of medical management and are reversible
	High	4	Causes health effects that are chronic, irreversible or fatal
Geographic extent	Localized	1	Effects occur in close proximity to UNG-Development and/or Production
	Community-wide	2	Effects occur across most of the community
Effectiveness of setback	Positive	1	Setback is anticipated to minimize health effects
	Negative	2	Setback is not anticipated to minimize health effects
Public health impact	Low concern	Green	Hazard received a score of 5–9
	Moderately high concern	Yellow	Hazard received a score of 10–14
	High concern	Red	Hazard received a score of 15–18

We summed scores to obtain an overall score for each hazard. These scores were then used to assign a qualitative ranking for the potential the public health impacts as follows:

H: High concern for potential of negative health impacts related to UNGDP (score of 15–18)
M: Moderately high concern for potential of negative health impacts related to UNGDP (score of 10–14).
L: Low level of concern for potential of negative health impacts related to (score of 5–9).

The overall rankings using our impact assessment methodology are presented in Table 2. Air quality, health care infrastructure, the social determinants of health and occupational health were ranked as HIGH Concern; water quality, noise, traffic and cumulative risk were ranked as MODERATELY HIGH Concern; and earthquake was ranked as LOW Level of Concern for their potential to negatively impact public health. Below we provide detailed descriptions of how we applied our impact assessment methodology for three hazards as illustrative examples, also summarized in Table 3.

**Table 2:** Overview of Hazard Ranking.

Evaluation Criteria	Air Quality	Water Quality	Noise	Earthquakes	Social Determinants of Health	Healthcare Infrastructure	Cumulative Exposure/Risk	Occupational
Presence of vulnerable populations	2	2	2	1	2	2	2	2
Duration of exposure	3	3	3	1	3	3	3	3
Frequency of exposure	2	2	2	1	2	2	2	2
Likelihood of health effects	3	1	2	0	3	2	2	3
Magnitude/severity of health effects	4	1	2	0	3	3	1	4
Geographic extent	1	2	1	2	2	2	2	2
Effectiveness of setback	1	2	1	2	2	2	2	2
Overall score	16	13	13	7	16	16	14	18
Public health impact	H	M	M	L	H	H	M	H

**Table 3:** Summary of Rationale for Hazard Ranking for Air, Water and Health Care Infrastructure Examples.

Evaluation Criteria	Score	Rationale for Score
Air Quality		
Presence of vulnerable populations	2	Individuals living closer to the UNGDP facilities will experience disproportionate exposure
Duration of Exposure	3	Exposure related to production, such as those associated with compressor stations will continue to persist for years/decades.
Frequency of exposure	2	Continuous exposure
Likelihood of health effects	3	Exposure to UNGDP related changes in air quality associated with adverse birth outcomes including NTD and CHD. Strong epidemiologic evidence from studies outside of UNGDP settings that show exposures to air pollutants associated with UNGDP related activities, including crystalline silica, VOCs, and PM have adverse health effects.
Magnitude/severity of health effects	4	Human studies from other fields show that exposure to air pollutants that are present in UNGDP processes are known to cause human health effects that can be irreversible, chronic, and at times fatal.
Geographic extent	1	Impact will be more pronounced in the immediate vicinity of the UNGDP facilities.
Effectiveness of setback	1	Adequate setback from the corner of a UNGDP facility to the corner of a residential property (2000 feet) can minimize exposure.
Water Quality		
Presence of vulnerable populations	2	Drinking water sources (well water) disproportionately contaminated near UNGDP facilities
Duration of Exposure	3	Exposure to contaminated water will exceed 1 year
Frequency of exposure	2	Exposure will be frequent
Likelihood of health effects	1	Despite presence of exposure, evidence regarding adverse health outcomes could not be determined because of insufficient data.
Magnitude/severity of health effects	1	Despite evidence of exposure, evidence regarding adverse health outcomes could not be determined because of insufficient data.
Geographic extent	2	Exposure can be widespread if the drinking water aquifer is contaminated
Effectiveness of setback	2	Setback will not mitigate exposure
Health Care Infrastructure		
Presence of vulnerable populations	2	Healthcare infrastructure disproportionately impacts those who are more likely to use healthcare services such as the elderly, the disabled, and children
Duration of Exposure	3	Exposure (the influx of UNGDP workers) will last for more than 1 year
Frequency of exposure	2	UNGDP worker health care utilization rates over the length of a UNGDP cycle will be constant
Likelihood of health effects	2	Stress on healthcare infrastructure will preclude individuals from receiving timely treatment
Magnitude/severity of health effects	2	Health infrastructure effects are noticeable but with proper management and resources, can be reversible
Geographic extent	2	Entire community is at risk
Effectiveness of setback	2	Adequate setbacks will not mitigate issues related to healthcare infrastructure

### Air Quality

Epidemiological studies over the past 50 years have documented the relationships between exposure to selected air pollutants (NOx, SOx, CO, ozone, particulate matter (PM), volatile organic compounds (VOCs), and polycyclic aromatic hydrocarbons (PAHs)) and various adverse health outcomes [14–22]. Recent data suggests these air pollutants are associated with UNGDP—some are produced as a part of the process (site preparation, production), while others are present in the natural gas. At present, linking exposure to air pollution associated with UNGDP—a new phenomenon—with adverse health outcome is challenging, first because of the discrepancy in temporal scale between onset of exposure (dating only few years back) and manifestation of outcomes that are known to have a notable lag time, particularly for chronic diseases, and second, because epidemiological studies designed to investigate such association are often 3–5 years in duration with additional 1–2 years for data to be published in a peer-reviewed journals. Despite these challenges, findings have started to emerge in peer-reviewed journals linking exposure to air pollution associated with UNGDP with increased risks of sub-chronic health effects, adverse birth outcomes including congenital heart defects (CHDs) and neural tube defects (NTDs) [23], low birth weight and preterm birth [23, 24], as well as higher prevalence of symptoms such as throat & nasal irritation, sinus problems, eye burning, severe headaches, persistent cough, skin rashes, and frequent nose bleeds among respondents living within 1500 feet of UNGDP facilities compared to those who lived >1500 feet [25, 26].

At present, there is a dearth of information that allows public health professionals to critically evaluate the relationship between exposure to air pollutants associated with UNGDP and health outcomes. Air samples collected within <0.5 miles of well pads during the well completion phase showed significantly higher concentrations of selected VOCs compared to samples collected more than 0.5 miles from the well pad (median concentration 2.6 vs 0.9 μg/m^3^, and 7.7 vs 4.0 μg/m^3^ for benzene and toluene, respectively)[25]. More relevant air pollution data for MD comes from a recent University of West Virginia study that showed considerably higher level of VOCs at monitoring sites located 625 feet from the well pad compared to the ones reported for Colorado, including benzene (mean 32.2 μg/m^3^, 95^th^ percentile 160 μg/m^3^), hexane (mean 10.4 μg/m^3^, 95^th^ percentile 22 μg/m^3^), acetone (mean 99.3 μg/m^3^, 95^th^ percentile 210 μg/m^3^). The concentrations of these VOCs in the West Virginia study varied considerably across different well pads. The WV study also collected air samples from control sites (Morgantown, WV) using an identical method. Although the sample size at the control site was limited (3), none of the control samples had detectable levels of VOCs.

#### Impact Assessment: Air Quality

Based on our evaluations of the limited but emerging epidemiological evidence from UNGDP impacted areas and air quality measurements as well as epidemiological evidence from other fields, we conclude that there is **a** **High Concern** UNGDP related changes in air quality will have a negative impact on public health in Garrett and Allegany Counties. The rationale used for scoring:

1. Vulnerable populations = 2. Concentrations of air pollution will decrease as the distance from the UNGDP facility increases. Therefore individuals living closer to the UNGDP facilities will experience higher exposures.
2. Duration of exposure = 3. While the exposure to air pollution resulting from site development may decrease once the site preparation is completed, exposures related to production, such as those associated with compressor stations will continue to persist for years/decades.
3. Frequency of exposure = 2. Indoor and outdoor exposure to air pollution occurs continuously, 24 hrs/day, 7 days/week, for individuals living in close proximity UNGDP activities.
4. Likelihood of health effects = 3. Emerging epidemiological evidence shows that exposure to UNGDP related changes in air quality may be associated with adverse birth outcomes including NTD and CHD. There is also strong epidemiologic evidence from studies outside of UNGDP settings that show exposures to air pollutants associated with UNGDP related activities, including crystalline silica, VOCs, and PM have negative effects on human health.
5. Magnitude/severity of health effects = 4. Exposure to air pollutants that are present in UNGDP processes are known to cause human health effects that can be irreversible, chronic, and at times fatal.
6. Geographic extent = 1. Impact will be more pronounced in the immediate vicinity of the UNGDP facilities.
7. Effectiveness of setback = 1. Prior evidence from traffic-related air pollution studies indicated that the concentrations of traffic-related pollutants drop to the background level beyond 500-700m (1640–2296 feet). Likewise, a study from Colorado reported air pollution levels significantly higher within 0.5 miles (2640 feet) of UNGDP facilities compared to >0.5 miles [23, 25]. Based on this, we concluded that an adequate setback from the corner of a UNGDP facility to the corner of a residential property (2000 feet) can minimize exposure.

### Water Quality

The water quality section includes an assessment of the impacts of UNGDP activities on water quality, soil quality, and naturally occurring radioactive materials (NORM).

The scientific literature has documented many plausible pathways by which natural and anthropogenic contamination may become available for human exposure as a result of UNGDP [27–31]. The evidence base to date suggests that gases, chemical compounds, and to a lesser extent naturally occurring radioactive materials (NORM), are mobilized during the drilling and wastewater recovery phases of the fracturing process and may result in contamination of ground waters used for drinking water [27, 30–35]. It is common for radium isotopes to be used as indices of radiological contamination, but emerging thought would suggest that radium alone might be an inadequate surrogate for monitoring radiological activity. Concerns also exist regarding the surface impoundment of wastewater in ponds or pits, in regards to both accumulation of radiological material and the concurrent potential for spills or leaks due to overfilling or ruptures in impoundment liners [28, 29, 36].

While challenges exist to assertions that fracturing activities affect drinking water sources, there appears to be scientific consensus that high-quality baseline and periodic monitoring data are largely absent in states that currently permit fracturing. This lack of data complicates assessment of the potential impacts of fracturing activities and may preclude determination of best practices or other interventions aimed at minimizing exposures. Despite these gaps, there is consistency in the literature that wells within shorter distances (typically <1 km) of drill sites are likely to be impaired, potentially by fracturing activities [37–39]. The most commonly documented contamination in these wells is methane gas.

Soil may be contaminated by drilling fluids, flowback, produced waters, and other wastes, which may contain numerous contaminants including radionuclides. Soil contamination is likely to occur through: 1) unintentional spills and leaks of waste or chemicals used during UNGDP, 2) the spread of waste onto fields, and 3) the use of wastewater or brine on roads. There is very limited information on how soil quality is impacted as a result of UNGDP. The few studies that do exist indicate an increase in calcium, magnesium, aluminum, manganese, zinc, chloride, sulfate, and sodium [40–42].

#### Impact Assessment: Water Quality

Based on our evaluations of the limited data available from UNGDP impacted areas, we conclude that there is a **Moderately High Concern** that UNGDP’s impact on water quality, soil quality and naturally occurring radioactive materials will have a negative impact on public health in Garrett and Allegany counties. The overall score for this hazard category is primarily driven by concerns related to water quality.

1. Vulnerable population received a score of 2 as exposure to contaminated water disproportionately affects residents near the UNGDP facilities, particularly those who rely on well water.
2. Duration of exposure received a score of 3 because exposure will persist for longer than 1 year.
3. Frequency of exposure received a score of 2 as exposure to contaminated water is frequent.
4. Likelihood of health effects was assigned a score of 1 because despite evidence of exposure, evidence regarding adverse health outcomes could not be determined because of insufficient data.
5. Magnitude/severity of health effects was assigned score of 1 because despite evidence of exposure, evidence regarding adverse health outcomes could not be determined because of insufficient data.
6. Geographic extent received score of 2 because exposure can be widespread if the drinking water aquifer is contaminated.
7. Effectiveness of setback was assigned score of 2 because setback will not mitigate exposure.

### Healthcare Infrastructure

A community’s healthcare infrastructure includes healthcare facilities (i.e., private and public healthcare services, hospitals, and emergency transport services) and adequately trained healthcare professionals. Allegany and Garrett counties have vast healthcare infrastructure needs as evidenced by their federally designations as Health Professional Shortage Areas (HPSA) and Medically Underserved Areas (MUA) with high levels of uninsured and medically assisted populations. UNGDP can have mixed impacts on a community’s health care infrastructure, especially one that is under-resourced. UNGDP revenues can fund improvements to the local healthcare infrastructure while at the same time, rapid population growth of UNGDP workers and families may intensify local health care utilization, overextending an already fragile system. [43, 44]

Data on the estimated number of UNGDP workers migrating into Allegany and Garrett Counties is unknown, but a recent economic impact assessment in Maryland approximated population growth and job growth based on low and high levels UNGDP development: a total of 8018 new residents under 25% development and 9422 new residents under 75% development during the first 10-year period of UNGDP; 1327–2825 new jobs on average during the first 10 years of drilling, and 151–189 new jobs on average during the 10-year period after drilling [45]. Information on whether UNGDP workers are adequately insured is also unknown; an impact assessment using case studies from Wyoming, Pennsylvania, and North Dakota indicates that the influx of uninsured and underinsured workers has had negative impacts on local healthcare infrastructure because of an increase in uncompensated emergency room visits [46].

This assessment is aligned with other studies which have indicated that workers in oil and gas industries experience seven times the fatality rate of general industry workers and non-fatal injuries and illnesses at higher rates than those in other industries [47–49]. Because of their exposure to higher rates of occupational related incidents and injuries, UNGDP workers may utilize emergency, urgent, and trauma care services at higher rates than the general population. Although UNGDP workers’ utilization rates may impact availability, access, and quality of healthcare services, very little data exists on utilization rates of industry workers and visitors and healthcare infrastructure impacts. Despite this gap, a handful of studies have suggested that workers place similar demands on health care infrastructure as local residents, with an increased demand on emergency department services [43, 44, 50].

#### Impact Assessment: Healthcare Infrastructure

Based on our review of limited literature on the impact of UNGDP on healthcare infrastructure, current predictions of the estimated workforce expected, and the health care infrastructure needs of Allegany and Garrett counties, we conclude that there is a **High Concern** that UNGDP related activities will have a negative impact on healthcare infrastructure.

1. Vulnerable population received a score of 2 as healthcare infrastructure impacts disproportionately those who are more likely to use healthcare services such as the elderly, the disabled, and children.
2. Duration of exposure received a score of 3 because exposure (the influx of UNGDP workers) will last for more than 1 year.
3. Frequency of exposure received a score of 2 as UNGDP worker health care utilization rates over the length of a UNGDP cycle will be constant.
4. Likelihood of adverse effect was assigned a score of 2 because stress on healthcare infrastructure will preclude individuals from receiving timely treatment.
5. Magnitude/severity of health effects was assigned a score of 2 because health infrastructure effects are noticeable but with proper management and resources, can be reversible.
6. Geographic extent received a score of 2 because the entire community is at risk.
7. Effectiveness of setback was assigned a score of 2 because adequate setbacks will not mitigate issues related to healthcare infrastructure.

## Discussion

The hazard ranking approach allowed us to systematically apply a set of evaluation criteria to each of the hazards identified through our literature review and scoping process. We applied the hazard ranking methodology to evaluate the hazards in terms of the likelihood to negatively impact public health (low concern, moderately high concern, and high concern). This approach allowed us to integrate the limited data and research along with the setback regulations to determine the extent of concern for each hazard.

There were some challenges applying the hazard ranking criteria to such a broad range of impacts, especially those that did not pertain to physical environmental hazards such as health care infrastructure. Healthcare infrastructure, the use of a community’s health care facilities and services, cannot easily be assessed according to our hazard ranking criteria. For instance, exposure in this scenario was not a chemical, biological, or physical hazard or a psychosocial stressor. Exposure was established as population influx, particularly migrant workers engaged in high-risk occupations, which we then determined to lead to increased demands on existing health care infrastructure. As a result, the effectiveness of setback had no bearing on healthcare infrastructure impacts and was determined not to mitigate issues related to healthcare infrastructure.

The report was presented to the Marcellus Shale Safe Drilling Initiative Advisory Commission, after a summary of the report was presented to the community in a public forum. While generally well received, comments received during and after the presentation suggested areas for improvement in future HIAs. First, since the cost-benefit analysis was done by a separate group prior to the HIA, we did not consider economic benefits in our report. This led some stakeholders, particularly those in favor of allowing UNGDP in Western Maryland, to question the objectivity of the process. In reality, our directive specifically excluded economic analysis. Future HIAs may benefit by having the two components together. Second, communities with whom we engaged were divided about the potential public health impacts of UNGDP. It may be beneficial to hold separate meetings or focus groups with different constituents during the scoping process. Since completion of our report, several other studies have identified the potential spread of endocrine disrupting chemicals (EDCs) in the aquatic environment related to fracking [51–53]. Future studies should consider EDC and their potential impact as a part of the monitoring plan, in addition to the ones identified in our recommendations. Finally, as mentioned above, the hazard ranking criteria utilized for evaluating the eight different topic areas are more applicable to environmental hazards. Applying these criteria towards other areas, particularly in the context of the healthcare infrastructure, posed challenges. For the sake of consistency, we used the same criteria for all topic areas, but this warrants additional investigation.

## Conclusion

UNGDP raises concerns about a range of potential human health, environmental, and socioeconomic impacts. Future sustainable development of land resources for energy requires a systems approach to account for potential impacts and trade-offs so that communities, local governments, and developers can be more fully informed in their decision-making and planning. In the absence of concrete data on exposure and adverse health outcomes surrounding UNDGP process, we developed a methodology to evaluate potential public health effects of different hazards using a consistent set of criteria. Our assessments of potential health impacts are not predictions that these effects will necessarily occur in Maryland, but instead provide an assessment of the impacts that could occur and need to be addressed by preventive public health measures if drilling is allowed. Should Maryland decided to move forward with UNGDP, our hazard ranking and overall report provided a set of recommendations that will minimize public health impacts. Our approach can be easily adapted by other communities facing similar situations as well as in other settings that entails making decisions with limited information.
